# Evaluation of physical education teaching effect using Random Forest model under artificial intelligence

**DOI:** 10.1016/j.heliyon.2023.e23576

**Published:** 2023-12-10

**Authors:** Xiaowei Jiang, Yuwei Du, Yingying Zheng

**Affiliations:** a College of Physical Education, Chengdu University, Chengdu, 610106, China; b School of Leisure Sport and Management, Chongqing University, Chongqing, 400044, China; c College of Physical Education and Health, Wenzhou University, Wenzhou, 325035, China

**Keywords:** Artificial intelligence, Random Forest model, PE teaching effect evaluation, Big data, Neural network

## Abstract

This work aims to optimize the physical education (PE) teaching effect based on deep learning (DL) to cultivate high-level college students better. Firstly, the present situation of college teachers' teaching ability is surveyed to realize the deficiencies in teaching. Secondly, an optimization algorithm is proposed to improve the node splitting mode. This algorithm can solve the problem of single and similar node splitting modes in the Random Forest (RF) algorithm. The independent node splitting method Iterative Dichotomiser 3 and Classification and Regression Tree in the algorithm are recombined, and new splitting rules are obtained through adaptive parameter selection. Finally, the scheme designed is tested. The results suggest: The results suggest: (1) During the training of the proposed algorithm, although the loss curve at 4550 and 6800 points has a small crest, the error of the network loss function shows a downward trend and tends to be flat; (2) Compared with unoptimized Genetic Algorithm (GA) and Genetic Algorithm-Back Propagation (GA-BP), the proposed algorithm shows better performance both in terms of time consumption and accuracy (time consumption is less than 5.4 ms, and accuracy is more than 95 %). In a word, using the GA-BP-RF algorithm proposed to improve the PE teaching effect is feasible. The proposed model provides ideas for applying DL technology to improve teachers' teaching abilities.

## 1 Introduction

With the rapid progress of society, the reform of quality education has attracted more and more attention. The reform of school physical education (PE) teaching is constantly exploring and advancing. Massive experts and scholars attach increasingly greater importance to the quality of classroom teaching. Smart teaching scheme refers to applying face recognition, gesture recognition, and online learning behavior analysis technology to traditional classroom and online learning to obtain students' attendance and learning status quickly. Then, an intelligent teaching system can be built to assist teachers and students in fully using the existing teaching resources and achieving ideal results quickly [1,2]. In the past decade, various sports activities have been constantly popularized in school PE teaching optimistically. More and more schools have begun to attach importance to the quality of PE classroom teaching, and the quality of classroom teaching needs to rely on special evaluation indicators. Consequently, it is crucial to construct indexes that conform to assessing the PE classroom teaching effect [3].

Artificial intelligence (AI) is currently a kind of widely used technology. There is a broad prospect for its development. Its appearance has greatly changed human life and industrial production, just like the application of steam engines, power systems, and the Internet. It has brought new development opportunities and directions to all walks of life and injected new vitality. Machine learning (ML) technology is to study the method of computer simulation or realization of animal learning behavior. Its purpose is to acquire new knowledge or skills, reorganize existing data structure, and make the program perform better. From the statistics perspective, ML is implemented by predicting data distribution and learning a model from the data. Then, the model is applied to the prediction of new data. Hence, it is required that test and training data have the same distribution. The primary feature of ML is to try to imitate the information transmission as well as processing mode between neurons in the brain. The most common is to apply it to computer vision and natural language processing (NLP). Random Forest (RF) is an integrated learning method combining the bagging method to generate multiple independent training sets and multiple classification regression trees for prediction. The highest or average voting scores determine the results. Its main idea is that the result of multiple classifiers is better than that of a single classifier. The problem studied here belongs to classification. RF algorithm is a commonly used algorithm to solve classification problems. RF is a kind of classifier using multiple trees for sample training and prediction [4,5]. RF algorithm is more easily accepted than a neural network, with higher accuracy, stronger robustness to noisy and missing data, and faster operation. Hence, it has a common application in data mining. For this reason, this work proposes an algorithm to optimize the node splitting method for the problem of single and similar node splitting methods in the RF algorithm. The independent node splitting method Iterative Dichotomiser 3 (ID3), and the Classification And Regression Tree (CART) in the algorithm are recombined. New splitting rules are obtained through adaptive parameter selection, used for selecting and dividing optimal attributes, and applied to image classification.

There are massive studies in related fields. Guo pointed out that college dance teaching was affected by multiple factors, and it was difficult to know how much each affects the teaching effect [6]. Data analysis and a decision tree (DT) model were adopted to overcome this difficulty to explore the factors affecting college dance teaching effect. First, the goals of college dance teaching for contemporary students were enumerated, and then the restrictive factors affecting the effect of college dance teaching were summarized. On this basis, an influencing factor analysis model was established, corresponding extensible DT was constructed, and verified through case analysis. Cui et al. proposed a new effect evaluation model for online teaching on the basis of Visual Question Answering (VQA). In this model, a guidance-attention model was proposed to discover guidance clues [7]. On this basis, key features were used to weight the model to achieve the location of the key information of the whiteboard and students' faces. Mao believed that the classroom was a vital communication environment in teaching activities, so schools and society should pay more attention to it [8]. However, in fact, the traditional teaching classroom relatively lacks communication. Facial expression recognition is a branch of high-precision facial recognition technology. Even in larger teaching scenes, it can also capture students' facial expression changes to analyze their attention accurately. Therefore, the evaluation of classroom teaching effects based on facial expression recognition was studied. Zhao argued that teachers were the theoretical pillars ensuring the college teaching level [9]. However, some colleges still lacked the support of data when evaluating teachers' teaching ability, leading to an evaluation process that is unreasonable as well as unscientific. Hence, teachers' teaching abilities cannot be truly and fairly reflected. A comprehensive college teachers' teaching ability evaluation model based on big data theory was first analyzed. Next, the effect after the model application was studied. Chen carried out an in-depth study on the fuzzy comprehensive evaluation (FCE) method and explored its application in PE teaching evaluation. Based on the primary deficiencies in PE teaching evaluation, a different PE teaching effect's evaluation system was built using the FCE method [10]. Yanru used AI for data analysis and adopted machine vision to identify the teaching process to assist PE teachers in evaluating PE teaching [11]. Nevertheless, there were still some deficiencies in the research of researchers. At present, scholars' studies on teachers' teaching effect evaluation mainly focus on the purpose, content, subject, method, and other aspects of the evaluation, while there is less research on the evaluation of the implementation procedure, mechanism, and students' learning effect. Some schools have some problems in the implementation, such as the utilitarian tendency of the evaluation purpose and the imperfect feedback mechanism. They only emphasize the role of student evaluation, ignoring the evaluation of students' learning effect.

In addition, Liu et al. proposed a time-contrast graph for self-supervised video representation learning. They introduced a new method for learning video representation using time-contrast learning and graph convolutional networks. They conducted a series of experiments to demonstrate the effectiveness of Tcgl in various video understanding tasks, highlighting its potential in self-supervised video representation learning [12]. Meng et al. put forward a study using a multilevel index system to assess the severity of online public opinion crises. They proposed a multilevel approach and discussed its implications for managing and responding to online public opinion crises [13]. Wu et al. introduced a product detection model for spam comments based on hybrid Positive-Unlabeled (PU) learning. This research discussed a new method for identifying spam comments in product review datasets using hybrid PU learning techniques. They demonstrated the effectiveness of their model in identifying deceptive reviews and debated its potential applications in improving the quality of reviews [14]. Chen et al. studied the continuation intention mechanism of middle school students on online learning platforms using qualitative comparative analysis. They explored the factors influencing middle school students' intention to continue using online learning platforms, providing insights into user behavior and platform design [15]. Cheng et al. introduced the study of context-aware dynamic service coordination in the Internet of Things (IoT) environment. They expounded on the challenges and solutions involved in coordinating services in an IoT environment. They proposed a context-aware dynamic coordination mechanism and provided insights on enhancing service coordination in IoT environments [16]. Lu et al. conducted a comprehensive review of attention mechanisms in multimodal fusion in VQA. They examined the various attention mechanisms used in the multimodal fusion of VQA, offering valuable insights into the latest techniques in the field [17]. Liu et al. presented an improved multi-label approach for sentiment classification of short texts. Their research focused on classifying sentiments in short text content and proposed an enhanced multi-label classification method. This research contributed to NLP and sentiment analysis [18]. Liu et al. described a semi-automatic method for developing a multi-label corpus of Twitter short texts. They outlined ways to create a corpus that can be used for various NLP tasks. This work helped provide tagged data for analyzing Twitter content [19]. Lu et al. viewed the extraction and fusion of multi-scale features of images and text in VQA. They explored techniques for extracting and combining features from image and text questions to improve VQA performance. Their research was conducive to improving the capabilities of VQA systems [20]. In addition, in AI, the Post-Quantum Cryptography (PQC) environment and its threats are also very concerning to researchers. For example, Sarker et al. studied error detection architectures applied to number theoretic transformations in hardware/software co-design methods. The study paid close attention to security issues in digital transformation in the new era of quantum computing, especially in the PQC environment. The research focused on improving the security of hardware/software co-design approaches to defend against potential threats and attacks, which were critical to protecting sensitive data and communications [21]. Kermani mentioned the issue of integrating emerging cryptographic engineering research with security education. His study highlighted the link between cryptography and security education to address evolving threats. In the context of PQC, enhanced security education was essential to train professionals to deal with future threats posed by quantum computing [22]. Mozaffari-Kermani studied high reliability and high-performance hardware architectures for Advanced Encryption Standard (AES) and Galois Counter Mode (GCM). This study emphasized the critical importance of hardware performance and reliability in modern communications and data security. The importance of this work was even more significant in the up-and-coming era of PQC, as traditional encryption methods needed to be evaluated and improved to meet future security needs [23]. Sarker et al. investigated efficient error detection architectures for Post-Quantum signatures Falcon's Sampler and KEM SABER. This research focused on enhancing the security of signatures and key exchanges in a PQC environment. Efficient error-detection architectures played a key role in defending against quantum attacks, so this research was crucial for developing the PQC field and responding to threats [24].

Moreover, in post-quantum computing and lightweight encryption, researchers also proposed some constructive studies on implementation and side-channel attacks to ensure the security of cryptographic systems. For instance, Cintas Canto et al. delved into the fact that algorithmic security was inadequate against actual attack threats in the Post-Quantum security domain. The study synthesized different implementation attacks, focusing on side-channel attacks, which may affect the security of PQC. The study called for more comprehensive research into implementation attacks and lightweight cryptography to ensure post-quantum security [25]. Azarderakhsh et al. reported the implementation of a hypersingular homomorphism Diffie-Hellman key exchange protocol on a high-performance Field Programmable Gate Array (FPGA). The work covered the areas of lightweight encryption and PQC, aiming to provide a more secure key exchange protocol. By implementing and optimizing this protocol, the research emphasized the importance of hardware implementation in protecting cryptographic systems [26]. Dubrova et al. described a side-channel attack against CRYSTALS-Kyber. This study highlighted the threat of side-channel attacks to PQC, especially for masked implementations. Besides, it also stressed the critical importance of implementing security against potential attack threats [27]. Kaur et al. conducted a comprehensive survey on the current NIST lightweight cryptographic standards' implementation, attacks, and countermeasures. The study emphasized the focus on lightweight encryption while highlighting the need to implement security to protect cryptographic systems from various attacks, including side-channel attacks. This study helped refine the implementation of lightweight cryptography to improve its resistance to potential attacks [28]. Finally, researchers did much work on Lightweight Cryptography (LWC) and building blocks. For example, Kermani and Azarderakhsh explored a reliable error detection architecture for Camellia block ciphers, suitable for alternative boxes of its different variants. The research focused on improving the reliability of cryptographic algorithms, especially in hardware implementations, to deal with potential errors. Through the application of an error detection mechanism, the research aimed to enhance the anti-interference and reliability of cryptosystems [29]. Cintas-Canto et al. presented the work on the first implementation of the NIST cryptography standard ASC0N and explored how Chat Generative Pre-Trained Transformer (ChatGPT) compares to lightweight cryptography. This study evaluated the performance differences between GPT models and ASC0N, particularly against lightweight cryptographic attacks. Through the study, the potential of the GPT model in terms of safety and performance can be better understood [30]. Kermani et al. pointed out security trends in embedded computing systems. The study introduced emerging security trends to the deep embedded computing system and provided a comprehensive overview of the field. This literature was a comprehensive effort to lead emerging security issues and solutions in embedded computing systems [31].

To sum up, with the rapid development of society, the reform of quality education and PE is gradually becoming the focus. However, there are still some challenges in assessing the quality of classroom teaching. Although face recognition, posture recognition, and online learning behavior analysis technology are introduced into the existing smart teaching programs, there are still shortcomings, especially in evaluating implementation procedures, mechanisms, and students' learning effects. At the same time, although AI and ML technology have made important progress in education, the evaluation of the effectiveness of PE teaching is still relatively limited. The existing research mainly focuses on the purpose, content, and method of evaluation, while the research on the procedure and mechanism of evaluation implementation is relatively insufficient. The comparison between previous studies and this work in various aspects is exhibited in Table 1.

**Table 1:** Differences between this work and previous studies.

Aspects	This work	Previous studies
Research focus	Teaching quality evaluation in information-based classrooms	A variety of topics, including dance teaching, online teaching, PE, etc.
Main methods	Comprehensive analysis, hierarchical structure, image classification	Data analysis, DT, visual elements, facial expression recognition, big data, FCE, etc.
Technology application	Image classification, informatization classroom evaluation, big data	VQA, machine vision, self-supervised learning, multilevel indicator system, NLP, etc.
Evaluation scope	Teaching quality evaluation in information-based classrooms	Teaching quality evaluation in multiple fields
Outstanding contributions	Teaching quality evaluation method based on information classroom	Introducing new assessment methods and techniques to provide research in different fields
Concerned problem	Teaching quality evaluation, machine learning, big data	Educational effect, user behavior, online review analysis, etc.

Therefore, from the perspective of AI, this work puts forward an innovative method to establish an evaluation index system of information-based classroom teaching quality, and implements a hierarchical structure model of each evaluation index through the analytic hierarchy process. In addition, aiming at the problem of node splitting mode in the RF algorithm, an optimization algorithm is proposed to improve node splitting mode, which recombines the independent node splitting mode of ID3 and CART to apply to the image classification problem. This work is novel in educational evaluation and ML. It is expected to offer an important reference and method for improving the accuracy and effectiveness of effect evaluation of PE teaching.

## 2 AI technology and improved RF algorithm

### 2.1 Research on neural network and Genetic Algorithm (GA)

**Fig. 1:**
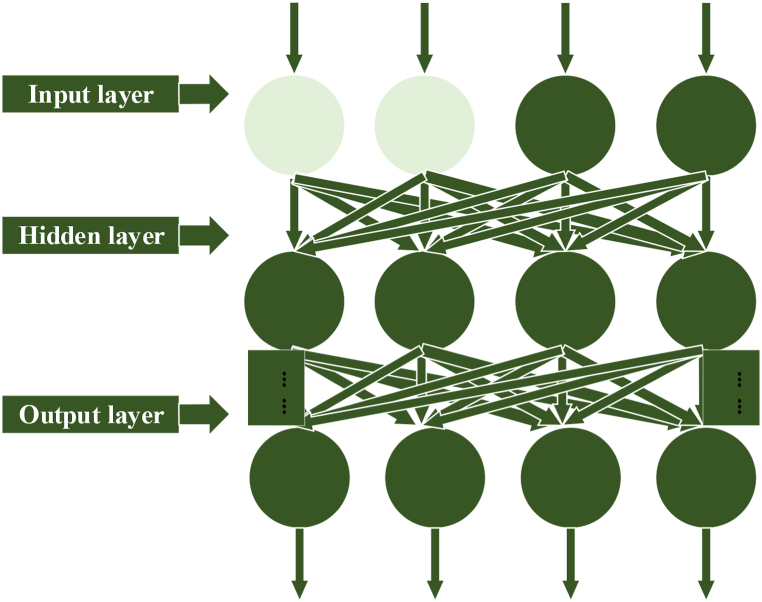
Structure of BPNN.

The neural network is a nonlinear system inspired by the human brain's nerves. It helps analyze data and information relationships based on sample data. Back Propagation Neural Network (BPNN) is a key deep learning (DL) model with strong prediction and problem-solving abilities. It comprises input, hidden, and output layers [[32], [33], [34]]. Input data passes through these layers to produce results; if errors occur, feedback is sent for correction. BPNN excels in prediction, self-improvement, fault tolerance, and generalization but has some uncertainty and sample selection requirements [35]. Fig. 1 shows BPNN's three-layer structure, enabling nonlinear mapping, parallel processing, distributed storage, fault tolerance, adaptability, and comprehensive reasoning. Patients provided verbal consent for anonymized information publication [36].

**Fig. 2:**
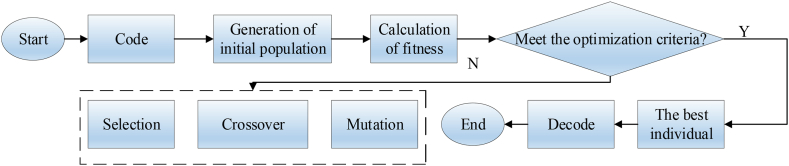
Flow of GA.

GA has a global search capability, is adaptable, highly customizable, and is easy to parallelize, which makes it ideal for optimizing neural network parameters. The global search nature and adaptability of GA help to find the global optimal solution in neural networks and improve the performance and generalization ability of the algorithm, especially for large-scale and complex tasks. Therefore, this work fuses the GA with the RF algorithm to enhance the model's performance. GA optimization is a process of iteration. It is implemented through coding, initial population generation, fitness calculation, genetic operation, and decoding. Binary encoding is an encoding method that has the widest application. Solutions are randomly selected, through which the initial population can be obtained. Generally, the larger the population is, the better the optimization effect is [37]. The specific genetic operation includes three steps. They are selection, crossover, and mutation. Fig. 2 presents the implementation of GA [38].

### 2.2 RF algorithm and improved RF algorithm

Both the DT algorithm and the RF algorithm are commonly used ML methods. DT algorithm builds a tree-like structure through gradual feature selection and segmentation of the data set. Each node represents a feature, each branch represents a judgment condition of a feature value, and the leaf node represents the final classification or regression result. Although DT is easy to understand and interpret and suitable for classification and regression tasks while being able to select important features automatically, it is prone to overfitting. Thus, techniques such as pruning are often required to optimize the model.

**Fig. 3:**
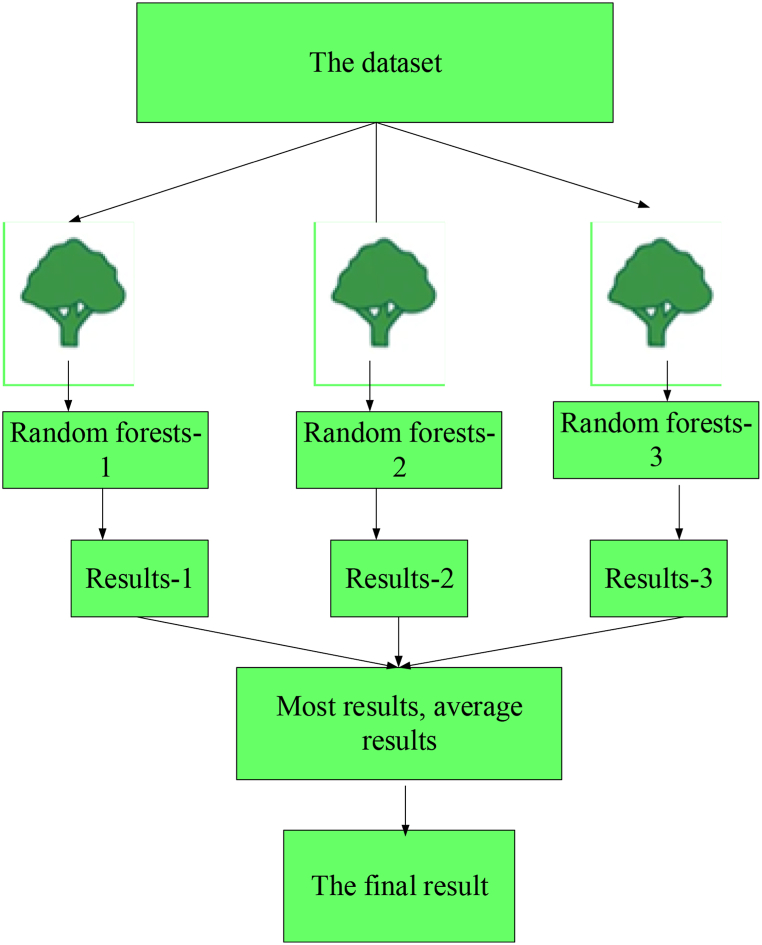
Flow of RF algorithm.

In contrast, the RF algorithm avoids overfitting. As a kind of combined classifier, RF extracts many samples from the primary samples using the Bootstrap resampling method to build sub-datasets. Next, a base DT is formed through the sub-datasets, and then trained. Random attribute selection was introduced into the DT training by RF. In other words, for each node of the base DT, a subset containing *k* attributes is selected randomly from the node's attribute set. Next, the best attribute is chosen from these subsets to be applied to the node splitting. In this way, each DT will differ, and the system's diversity will be improved. Then, these DTs are combined, the samples not extracted in Bootstrap are used as a reference for verification, and the classification results are obtained through voting [[39], [40], [41]] to improve the classification performance. Fig. 3 denotes the flow chart of the algorithm.

Fig. 3 shows that the RF algorithm contains three key branches: Bootstrap resampling, attribute selection, and voting. Bootstrap resampling introduces randomness, builds multiple sub-datasets, and increases model diversity. Attribute selection introduces random attributes into node splitting to improve model generalization ability. The voting method integrates the results of each DT, reducing errors, and these branches work together to make RF a powerful ML algorithm.

In the RF algorithm, node splitting is the most crucial step. Only through node splitting can a complete DT be generated. Each tree branch is generated by selecting attributes according to several splitting rules. The primary rules are the principles of maximum information gain, maximum information gain ratio, and minimum Gini coefficient. Next, an attribute is selected as a split attribute, and the DT branch will grow based on its division. After the continuous development of the division process, the purity of the node is getting increasingly higher. It means that the samples in the node belong to the same category as far as possible. From massive research results, it can be obtained that the RF algorithm performs well in classification. It has higher accuracy and an excellent tolerance for abnormal values and noise. Moreover, the algorithm is not prone to overfitting. The improved RF algorithm can optimize the node-splitting algorithm of the DT through the parameters' adaptive selection process to enhance the algorithm's classification accuracy. For the same dataset, if different node splitting algorithms are selected, different DTs will be obtained due to different selected attributes, and the classification accuracy of RF will be different. Hence, it is proposed to select the optimal attribute for node splitting when generating the DT. It means the node splitting algorithm is linearly combined to form new splitting rules applied to selectively partition node attributes. Since the integrated node splitting algorithm in Spark mllib's RF algorithm is only ID3 as well as CART, its node splitting equation represents the information gain as well as the Gini coefficient obtained by dividing the sample set D with attributes, as shown in equations (1), (2):

(1) $$Gini\left(D,a\right)=Ent\left(D\right)-\sum_{v=1}^{V}\;\frac{\left|D^{v}\right|}{\left|D\right|}\text{Ent}\left(D^{v}\right)$$

(2) $$Gini\left(D,a\right)=\sum_{v=1}^{V}\;\frac{\left|D^{v}\right|}{D}Gini\left(D^{v}\right)$$

In equations (1), (2), $D^{v}$ represents all samples with a value of $a^{v}$ on attribute a in D contained in the v-th branch node.

(3) $$Ent\left(D\right)=-\sum_{k=1}^{\left|y\right|}\;p_{k}\;\log_{2}p_{k}$$

(4) $$Gini\left(D\right)=\sum_{k=1}^{\left|y\right|}\;\sum_{k^{\prime }\neq k}\;p_{k}p_{k}^{\prime }=1-\sum_{k=1}^{\left|y\right|}\;p_{k}^{2}$$

Equations (3), (4) represent dataset *D*'s information entropy and Gini value.

The node splitting criteria should aim at higher purity of the dataset after partitioning. Hence, the combined node splitting equations (5), (6) are as follows:

(5) $$H=\min_{\alpha ,\beta \in R}\;F\left\{D,a\right\}=\alpha \text{Gini}\left(D,a\right)-\beta \text{Gain}\left(D,a\right)$$

(6) $$s.t.\;\left\{ \begin{array}{c} \alpha +\beta =1\\ 0\leq \alpha ,\beta \leq 1 \end{array}\right.$$

The parameters α and β represent the coefficients of the H(x) function, where the value of H is the minimum; ID3 and CART are both optimal as the node splitting criteria to enhance the classification effect [[42], [43], [44]].

### 2.3 The design and solution of the evaluation index of informatization teaching quality

**Fig. 4:**
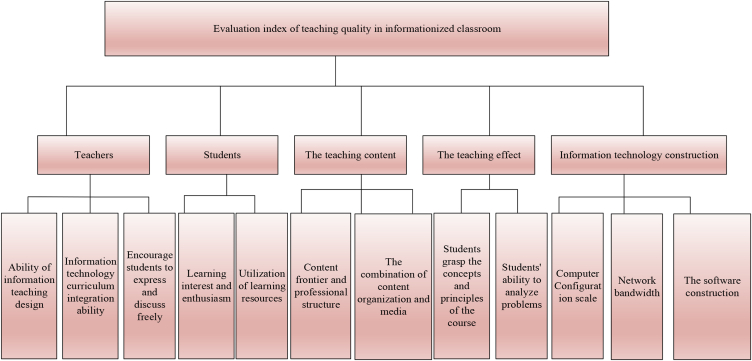
The evaluation index system of teaching quality.

An evaluation index system for the information classroom's teaching quality is established through the above indexes. The evaluation index system of teaching quality is drawn in Fig. 4.

In Fig. 4, information technology (IT) architecture plays a crucial role in education, encompassing digital course materials, online learning platforms, student information systems, etc. These tools and resources are essential to support the educational process and enhance teaching quality. Modern education relies on the support of IT, and schools and educational institutions need to build a strong IT infrastructure to meet educational needs. Hence, IT architecture, as a key aspect of education quality, cannot be ignored. It can affect the teaching effect of teachers, the learning experience of students, and the overall education quality. At present, a variety of studies have emphasized the importance of IT architecture for teaching quality. For example, Robinson (2019) pointed out that in modern higher education, effective IT architecture can improve students' learning outcomes and satisfaction [45]. In addition, Papay et al. (2022) studied the relationship between the teacher evaluation model and IT architecture. They found that IT architecture significantly impacted teacher evaluation results [46]. These findings highlighted the importance of IT architecture in the education sector and suggested that it can be used as a key index for teaching quality evaluation.

This work adopts AHP to decide the weight of the information classroom teaching quality evaluation indexes. The process is described in detail as follows.

The first step is to implement a hierarchy model. The basic task of AHP is to implement the hierarchical structure model. Consequently, the primary task in evaluating the classroom teaching quality is establishing the evaluation index level of information classroom teaching.

The second step is to determine the judgment matrix. In the evaluation index of informatization classroom teaching, two indicator factors of the same level are selected and compared with the 1–9 scale method to explore their importance. For indicator factor j, the importance of the factor i on it is determined according to the value of $a_{ij}$. A refers to the judgment matrix, which has n orders. Its expression in equation (7) as follows:

(7) $$A=\left(\begin{array}{cccc} a_{11} & a_{12} & \cdots & a_{1n}\\ a_{21} & a_{22} & \cdots & a_{2n}\\ \vdots & & & \vdots \\ a_{n1} & a_{n2} & \cdots & a_{nn} \end{array}\right)$$

λ is the eigenvalue of the judgment matrix. Moreover, it should satisfy the condition Aw−λEw=0. *w* refers to the eigenvector of the judgment matrix. If λ is $\lambda_{\max}$, the equations will be acquired. The solution vector of the equation set is solved to determine each indicator factor's weight, that is, $W=\left(w_{1},w_{2},\cdots ,w_{n}\right)$.

The third step is to check the matrix consistency. C⋅R is the consistency ratio, and the matrix consistency can be verified through its value. In the verification of the consistency of the judgment matrix, the condition of C⋅R small enough must be satisfied, that is, C⋅R⩽0.1. In this way, a better hierarchical single-sorting effect can be obtained.

When the C⋅R conditions cannot be satisfied, the judgment matrix shall be adjusted until the conditions are met. The verification procedures are described in detail as follows.

1) The consistency indicator is determined. Its expression can be written as equation $C\cdot I={\left(m-1\right)}^{-1}\;\left(\lambda_{\max}-m\right)$.
2) The average random consistency indicator R⋅I can be acquired by querying.
3) CR is solved. The solution equation is $C\cdot R=C\cdot I*{\left(R\cdot I\right)}^{-1}$. If C⋅R⩽0.1, A is used as the judgment matrix. If not, it is essential to readjust the judgment matrix.

The weight of the classroom teaching quality evaluation index can be solved in two steps.

1) Solving the primary indicator weight. In the hierarchical structure of the informatization classroom's teaching quality evaluation, the primary indicators are teachers, students, teaching content, teaching effect, and informatization construction. Questionnaires, expert opinions, and other methods are adopted to determine the judgment matrix for evaluating information classroom teaching quality, and to test its consistency. When the test is successful, under the condition of the maximum eigenvalue of the matrix, its corresponding eigenvector is solved, and $W=\left(w_{1},w_{2},w_{3},w_{4},w_{5}\right)$ is the first-level weight vector.
2) Solving the second-level index weight. Several second-level teaching quality evaluation indexes are adopted to describe the first-level indexes in this evaluation system.

**Fig. 5:**
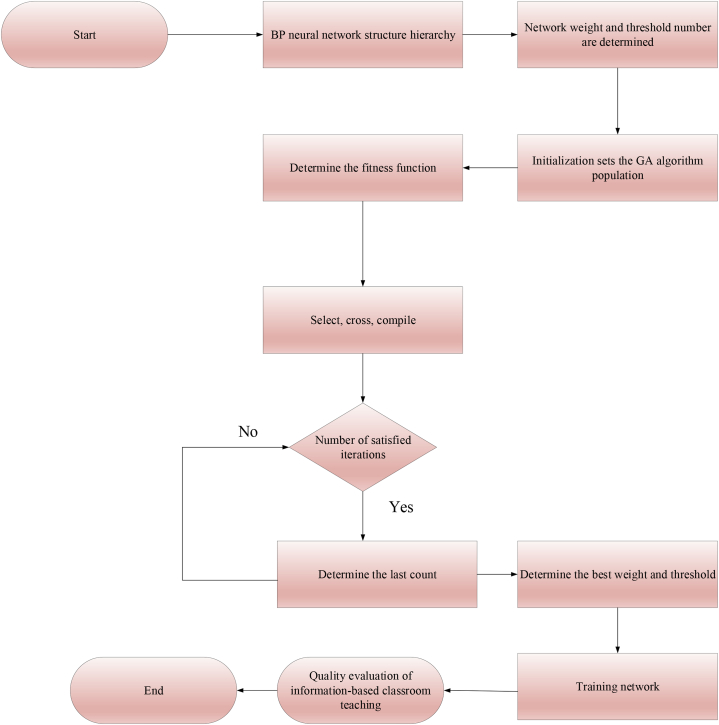
Evaluation model of informatization classroom teaching quality.

The best input weight and the threshold value of BPNN are determined through GA, so BPNN has high accuracy in evaluating information classroom teaching quality. Fig. 5 signifies the construction process of the GA-BP-based informatization classroom teaching quality evaluation model.

### 2.4 The Genetic Algorithm-Back Propagation-Random Forest (GA-BP-RF) algorithm

**Fig. 6:**
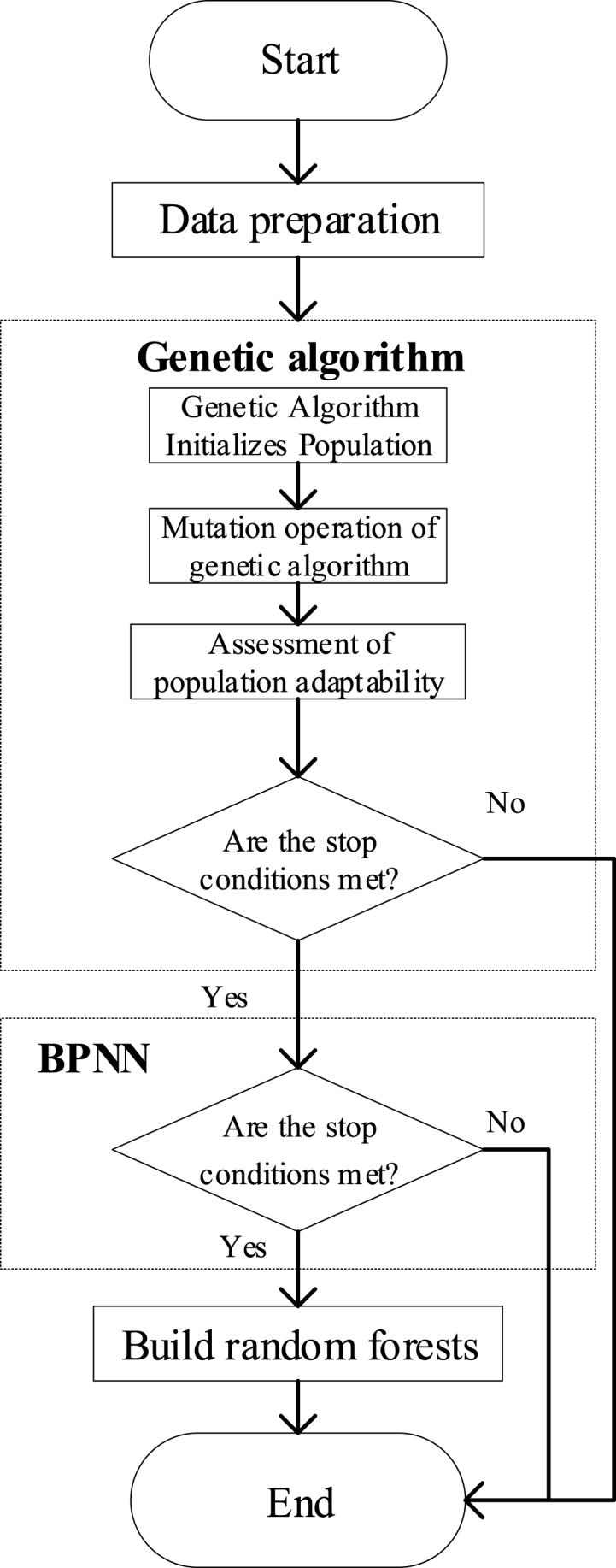
Implementation flow of the GA-BP-RF algorithm.

According to the above sections, this work constructs a new GA-BP-RF algorithm based on GA-BP, and the algorithm flow is plotted in Fig. 6.

Fig. 6 demonstrates the flow of GA-BP-RF algorithm as follows. First, the weights and biases of the neural network are initialized and optimized by GA, and then the BP algorithm is used to train the neural network to improve accuracy. Next, the RF is built to integrate multiple DTs, and each DT is trained based on a random attribute selection. Lastly, the neural network and RF output are integrated to generate the final prediction results. This combined algorithm makes full use of the optimization power of GA, the learning power of neural networks, and the stability of RF and is suitable for various ML and data analysis tasks.

### 2.5 Experimental environment configuration

Table 2 exhibits the parameter setting of the hardware and software used in this experiment.

**Table 2:** Configuration of the experimental environment.

Parameters of hardware platform	Parameters of software environment
Intel Core I7–6700K Quad-core 8-threaded processor	Ubuntu 16.04 Keras 2
Nvidia GTX X video card	CuDNN 7.4, CUDA 9.0

## 3 Result analysis and discussion

### 3.1 DL algorithm test

**Fig. 7:**
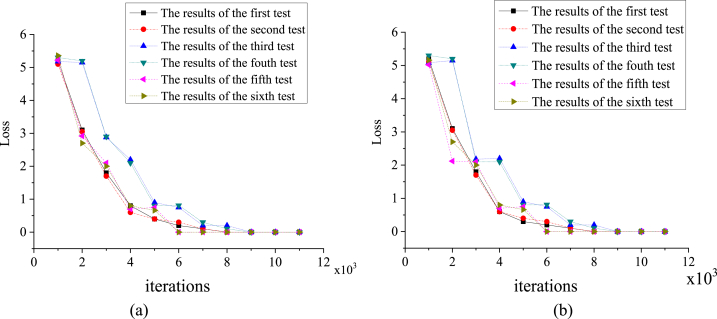
Variation curve of the network error (a. scenario 1; b. scenario 2).

This section explores the training of the RF algorithm before and after optimization. The dataset selected in the experiment is from the Scenario-Based RF Training Data set. Two scenarios are set up during the experiment, corresponding to the RF algorithm before and after optimization. Then, six loss cases of the two algorithms are obtained by setting different learning rates for the algorithm (0.001, 0.002, 0.003, 0.004, 0.005, and 0.006). In addition, the algorithm's loss function and optimization settings are consistent: the Sigmoid function and Adam optimizer, respectively. Fig. 7(a) and (b) portray the error change curve of the RF algorithm without improvement and the improved RF algorithm.

The training process of the improved algorithm shows that when the number of iterations reaches 3500, although the loss curves at 4550 and 6800 have small peaks, the overall error of the network loss function presents a downward trend and tends to be flat. After 8000 iterations, the loss function error of the network is basically stable below 0.2, and gradually tends to be stable. The original algorithm's training process shows that the network's loss function error is stable when the number of iterations reaches 7800, below 0.25. The training error of the unimproved network is always higher than that of the improved network, and the training effect of the improved network model is superior to that of the original network model.

### 3.2 System test results

**Fig. 8:**
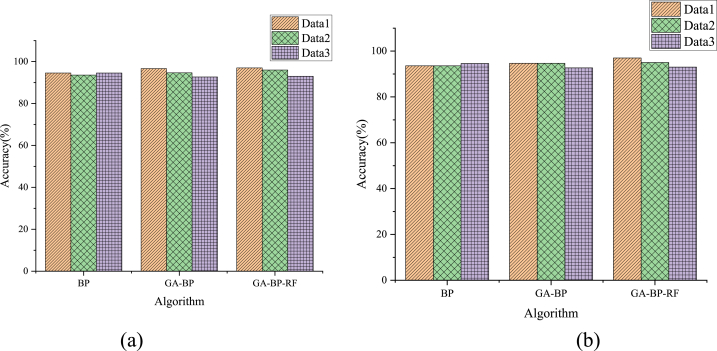
Comparison of algorithm accuracy ((a) simple data; (b) complex data).

**Fig. 9:**
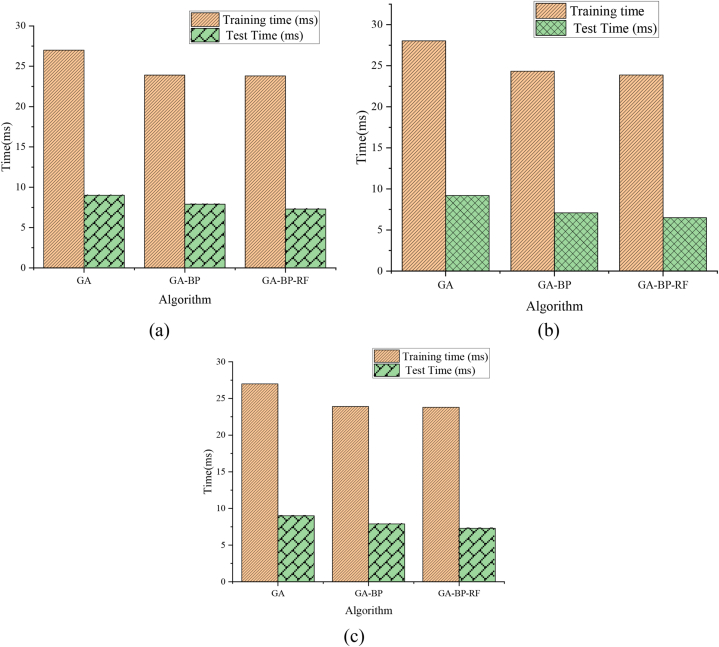
Comparison of algorithm consumption time ((a) Data 1; (b) Data 2; (c) Data 3).

BP, GA-BP, and GA-BP-RF algorithms categorize the experimental data in the teaching platform's performance evaluation scale. Fig. 8(a) and (b) display the accuracy of the BP, GA-BP, and GA-BP-RF algorithms. Fig. 9(a) and (b) show the algorithm testing and training time. In the figure, simple and complex data represent two data sets of different complexity in the Scenario-Based RF Training Data set (divided according to data complexity). Data 1, 2, and 3 refer to the three sub-data sets in simple and complex data.

Fig. 8(a) and (b) suggest that the GA-BP-RF algorithm is superior to the BP and GA-BP algorithms in prediction accuracy. The primary reason is that the GA-BP-RF algorithm generates a Complete Binary Tree. Hence, the classes that are easy to partition are divided first, avoiding error accumulation and improving the division accuracy.

Fig. 9(a) and (b) depict that when testing Data 1, 2, and 3, the GA algorithm consumes more time than the GA-BP and GA-BP-RF algorithms. GA-BP and GA-BP-RF algorithms consume almost the same time in the classification process. However, the difference between the GA-BP and the GA-BP-RF algorithms will be greater with the increasing amount of data. In a word, the GA-BP-RF algorithm proposed here is feasible to assess the quality of college classroom teaching.

### 3.3 The performance analysis of algorithms

**Fig. 10:**
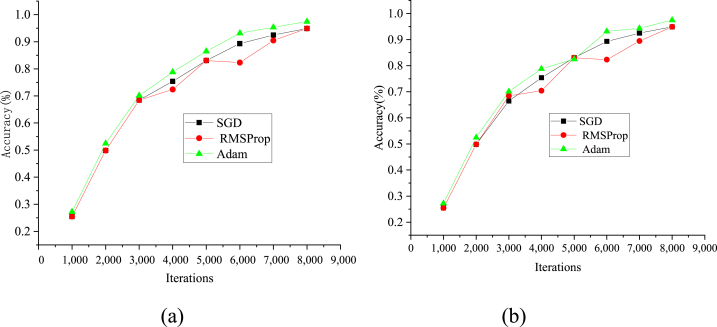
Comparison of different optimization algorithms ((a) results of the first test (b) results of the second test).

Different optimization algorithms have various effects on the model. Here, Stochastic Gradient Descent (SGD), Root Mean Square Prop (RMSProp), and Adam are used in experiments to compare their effects on the model. The result is represented in Fig. 10 (a) and 10(b).

Fig. 10(a) and (b) reveal that these optimization methods have a similar effect. The difference is that the Adam method converges faster than RMSProp. SGD is faster, but its accuracy is unstable and fluctuates. Hence, after comprehensive consideration, the Adam algorithm is selected as the optimization algorithm.

Moreover, a confusion matrix is introduced to more fully evaluate the performance of the improved model. The confusion matrix is an important tool for measuring the classification accuracy of a model, including the number of true positives, true negatives, false positives, and false negatives. The data set used here comes from multiple college PE courses and contains 400 sample data, including students' physical test scores, class participation, academic scores, and other information. For experiments, the data set is divided into two parts: training set and verification set, in which the number of training sets is 320 and the number of verification sets is 80. The final experimental results are outlined in Table 3.

**Table 3:** Results of model performance evaluation.

Dataset	True positives	True negatives	False positives	False negatives	Selectivity	Sensitivity	F-score
Training set (The proposed algorithm)	320	420	30	20	0.933	0.941	0.937
Untrained set (The proposed algorithm)	80	100	8	12	0.926	0.870	0.897
Training set (Traditional algorithm)	320	380	70	60	0.844	0.824	0.834
Untrained set (Traditional algorithm)	80	90	18	22	0.833	0.761	0.796

Table 3 describes that the proposed algorithm has a high number of true positives and negatives on both the trained and untrained sets, indicating that the model has many correct classifications on the positive and negative categories, especially on the training set. Besides, the relatively low number of false positives and negatives indicates that the model has a lower error rate on the training data, but a slight increase on the untrained set. The selectivity and sensitivity are close to 1 on the training set, showing that the model has high classification accuracy on the trained data, while the selectivity is slightly decreased but still acceptable on the untrained set. The F-score, which considers selectivity and sensitivity, performs well, especially on the training set. However, it remains relatively high on the untrained set, illustrating that the model performs well on new data classification. These results denote that the improved GA-BP-RF algorithm has a good classification effect in PE teaching cases and has the potential to be applied to practical education scenarios.

### 3.4 Evaluation effect of the GA-BP-RF algorithm on PE teaching

In this section, the application effect of the GA-BP-RF algorithm in evaluating the teaching quality of college PE classroom informatization is verified. Data on student and teacher interaction from different PE classrooms, including PE curriculum content and physical performance, were collected for this purpose. The experiment was classified into the experimental and control groups, in which the GA-BP-RF algorithm was utilized to evaluate the quality of information teaching in the experimental group, and the traditional method was used in the control group. The main indicators of concern are the accuracy and efficiency of the evaluation results and the overall performance of PE teaching. Through the above experiments, the final experimental results are detailed in Table 4.

**Table 4:** Application effect of the GA-BP-RF algorithm in the evaluation of information teaching quality in PE classroom.

Student number	Experimental group score	Control group score
1	0.92	0.75
2	0.88	0.71
3	0.94	0.68
4	0.90	0.73
5	0.95	0.70
6	0.91	0.72
7	0.89	0.69
8	0.93	0.74
9	0.87	0.67
10	0.96	0.76
Average score	0.91	0.68
Evaluate efficiency	35 min	50 min

Table 4 manifests that the average score of students in the experimental group using the GA-BP-RF algorithm for evaluation is 0.91, while the average score of students in the control group employing traditional methods is 0.68. This indicates that the evaluation results of the experimental group are significantly higher than those of the control group, illustrating that the GA-BP-RF algorithm has higher accuracy in evaluating the quality of PE teaching. Furthermore, the evaluation efficiency of the experimental group is also higher, taking only 35 min compared to 50 min in the control group. Therefore, the GA-BP-RF algorithm improves the evaluation accuracy and efficiency, which is suitable for the PE teaching field and is expected to advance the teaching quality and effect.

### 3.5 Discussion

The GA-BP-RF algorithm is adopted in this work, which combines the topological structure of GA, BP algorithm, and RF to be used in the data classification task in PE. GA is employed to optimize the weights and biases of the neural network, including population size, crossover probability, mutation probability, and other parameters. The BP algorithm establishes a multi-layer feedforward neural network. The number of hidden layer neurons can be adjusted according to the complexity of the problem, and the Sigmoid activation function is used to realize nonlinear mapping. The RF consists of multiple DTs, each built on a randomly sampled sub dataset, with an adjustable number and depth of trees. Experimental results show that the improved RF algorithm has significant advantages in the training process. The training error gradually decreases and tends to be flat after 3500 iterations. In contrast, the original algorithm tends to be stable only after 7800 iterations. Moreover, the training effect of the improved network model is obviously better than that of the unimproved network model. After comparing the performance of BP, GA-BP, and GA-BP-RF algorithms in the system test, it is found that the GA-BP-RF algorithm presents high prediction accuracy. This is mainly because it uses the strategy of generating a complete binary tree, avoids the accumulation of errors, and improves the partition precision. The GA-BP-RF algorithm has a slight increase in processing time, especially when dealing with big data, and the difference between it and the GA-BP algorithm will be more remarkable. In general, the proposed GA-BP-RF algorithm is feasible. It performs well in evaluating the quality of university classroom teaching. This work comprehensively considers SGD, RMSProp, and Adam optimization algorithms in the optimization algorithm selection. The results reveal that the Adam algorithm is evidently superior to RMSProp in convergence speed and has better accuracy and stability, so it is selected as the optimization algorithm. To sum up, the GA-BP-RF algorithms have a wide range of applications in education, big data analytics, data mining, environmental monitoring, and healthcare, where they can be used to improve education quality, optimize decision-making, improve predictive models, and promote innovation.

## 4 Conclusion

In this work, a GA-BP-RF algorithm is proposed, which combines GA, BPNN, and RF to evaluate the performance of educational platforms and study DL problems. Firstly, the GA-BP-RF algorithm is designed and implemented, which fully utilizes the optimization ability of GA, DL characteristics of BP, and the diversity and robustness of RF. Thus, it can perform well in education quality evaluation and DL problems. By using GA to adjust the parameters of BPNN, the model's performance is improved, and its robustness and generalization ability are enhanced by combining the characteristics of RF. Secondly, the system test is carried out in the education sector. The GA-BP-RF algorithm is applied to the performance evaluation of the education platform and compared with the traditional BP algorithm and GA-BP algorithm. The experimental results reveal that the GA-BP-RF algorithm is superior to the other two algorithms' prediction accuracy, especially in processing complex data. In addition, different optimization algorithms (SGD, RMSProp, and Adam) are also compared. The results show that compared with the unoptimized GA algorithm and GA-BP algorithm, the proposed algorithm performs better in terms of both time consumption and accuracy (time consumption is less than 5.4 ms, accuracy is greater than 95 %). Then, the GA-BP-RF algorithm is compared with the traditional forward propagation scheme, and the results indicate that the GA-BP-RF algorithm performs well in the performance indexes of true positive, true negative, false positive, false negative, selectivity, sensitivity, and F-score on both the training set and the untrained set. Finally, the actual teaching evaluation effect of the GA-BP-RF algorithm is verified, and the verification results demonstrate that this algorithm has higher accuracy in the evaluation of PE teaching quality, and the evaluation efficiency only takes 35 min, while the traditional evaluation method takes 50 min. However, there are some limitations. First, although the GA-BP-RF algorithm has achieved satisfactory results in education and DL, it has high computational complexity and requires longer training time. Moreover, this work covers only a limited number of applications, and its applicability in other fields has not been widely verified. Future research directions include further optimization of algorithm performance, reduction of computational complexity, and wide application of the GA-BP-RF algorithm in more fields. In addition, attempts will be made to explore combining this algorithm with other ML and DL techniques to improve the performance and diversity of the model. In conclusion, the GA-BP-RF algorithm has the potential to be extensively applied in the education and DL fields. Despite some limitations, further research and improvement are believed to bring more innovation and progress to these fields. In addition, on the one hand, future research can focus on the application of the GA-BP-RF algorithm to more practical scenarios, encompassing data mining, NLP, image processing, etc. This will help expand the application of algorithms, provide more solutions, and drive innovation and progress. On the other hand, in the future, this work will focus on improving the robustness and security of the GA-BP-RF algorithm to prevent potential attacks and risks. This could include work on adversarial training, privacy protection, and security analysis.

## CRediT authorship contribution statement

**Xiaowei Jiang:** Conceptualization, Data curation, Formal analysis, Methodology, Writing – original draft. **Yuwei Du:** Methodology, Formal analysis, Software. **Yingying Zheng:** Writing – review & editing, Validation, Methodology, Investigation, Data curation.

## Funding statement

This research was supported by the project of “Research on the Talent Training Model for Interdisciplinary Sports Master's Students”, The 14th Five-Year Plan Graduate Teaching Reform Project of Zhejiang Province in 2022 (Grant no.: Zhejiang Position Office [2023] No. 1, Serial number 343).

## Declaration of competing interest

The authors declare that they have no known competing financial interests or personal relationships that could have appeared to influence the work reported in this paper.
